# One Step Ahead

**Published:** 1880-03

**Authors:** 


### ONE STEP AHEAD.

At a recent meeting of the "Pittsburgh Dental Association," the following was unanimously adopted:

WHEREAS. The profession of Dentistry now suffers, and has suffered in the past from the empiricism of many of its practioners, and

WHEREAS. The Dentist occupies the position of a public teacher; his education should be such as to enable him to give a satisfactory and scientific reason for what he does, or proposes to do, therefore

*Resolved.* First, that an applicant for the position of student in the office of any member of this Association, shall not be less than eighteen years of age, be of good moral character, and shall furnish satisfactory testimonials of the same.

SECOND. A diploma from any Chartered Literary Institution, shall be deemed sufficient evidence of an applicant's preliminary educational qualifications.

THIRD. The minimum preliminary educational qualifications shall be thorough knowledge of Orthography, Reading, Writing, Arithmetic, Grammar and Geography, together with a knowledge of the Latin Grammar, and ability to read and translate a selection from any of the first five books of Cæsar's Commentaries.

FOURTH. That an Examining Board, composed of three active members of this Association, be elected at each annual meeting. The duty of said Board, shall be to examine all applicants for admission, as students, to members of this Association. After examination of an applicant, each member of this Board, will be entitled to ten votes, the applicant to receive twenty votes to entitle him to become a student.

W. F. FUNDENBERG, M. D., Pres't.

H. W. ORR, D. D. S., Sec'y.

This is a higher standard for the preliminary qualification of dental students, than has been required or proposed by any other Dental Association, or than by any College in this country, and yet neither the profession nor the colleges should require anything less than this, but more just so soon as practicable.

Medical Literature, from which the dental student must draw much of his professional food, is crowded with a nomenclature from other languages, chiefly from the Latin and Greek; some knowledge of these should be possessed by any dental student, in order that he may easily and intelligently study the various branches of his course, without some knowledge of these languages, he is in-about the position of the pupil who would attempt to solve the problems of higher arithmetic without a knowledge of the mutliplication table.

The Dental Profession; our Associations and all our Colleges should take a much higher position than they now occupy, in regard to the preliminary education of those who are hereafter to enter its ranks. The Pittsburgh Society has shown how this may be done.

